# Supplementary material for: Integrated Metabolomics and Lipidomics Analysis Reveals the Mechanism Behind the Action of Chiglitazar on the Protection Against Sepsis-Induced Acute Lung Injury
Source: Metabolites. 2025 Apr 25;15(5):290. doi: 10.3390/metabo15050290 (PMC12112857; doi:10.3390/metabo15050290)
Supplement: Supplementary file 1 [file metabolites-15-00290-s001.zip › metabolites-3579937-supplementary.pdf]

<u>SUPPLEMENTAL INFORMATION:</u>

Integrated metabolomics and lipidomics analysis reveals the mechanism behind the action of chiglitazar on the protection against sepsis-induced acute lung injury

Liu-Liu Lu[2#], Yu-Li Cao[3#], Zhen-Chen Lu[2], Han Wu[2], Shan-Song Hu[2], Bing-Qing Ye[2], Jin-Zhi He[2#]， Lei Di[2], Xu-Lin Chen[4*], Zhi-Cheng Liu[1,2*]

1. The Third Affiliated Hospital of Anhui Medical University, The First People's Hospital of Hefei, 230071, China
2. School of Pharmacy, Anhui Provincial Laboratory of Inflammatory and Immunity Disease, Anhui Institute of Innovative Drugs, Anhui Medical University, Hefei, 230032, China
3. The first department of critical care medicine of the second affiliated hospital of Anhui Medical University, Hefei, 230601, China
4. Department of Burns, the First Affiliated Hospital of Anhui Medical University, Hefei, Anhui 230022, China

#: these authors contributed equally to the manuscript.

***: Corresponding author:**

Pr. Xu-Lin Chen, e-mail address: okcxl@126.com,

Dr. Zhicheng Liu, e-mail address: liuzhicheng@ahmu.edu.cn.

Supplemental Figure S1

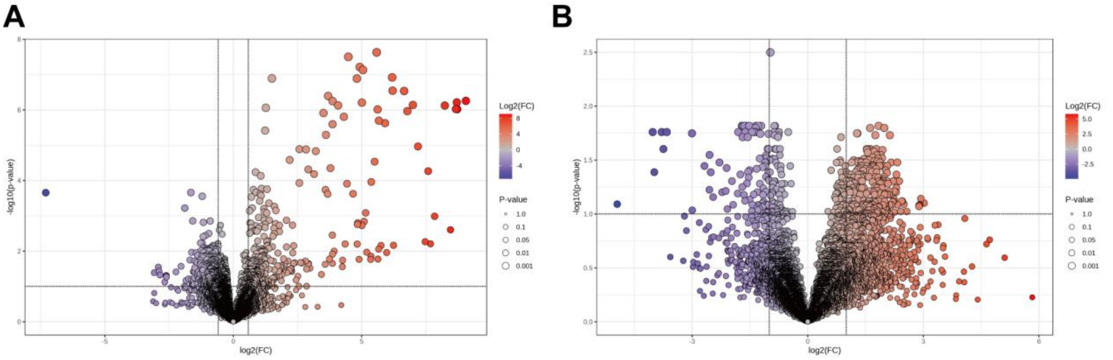

Figure S1: Volcano plots analysis in metabonomics. (A) CLP vs. Sham volcano plots analysis. (B) CLP+Chi 10 vs. CLP volcano plots analysis. Wherein, “S” represents the sham surgery group; “M” represents the CLP group; “D” represents the CLP+Chi 10 group.

Supplemental Figure S2

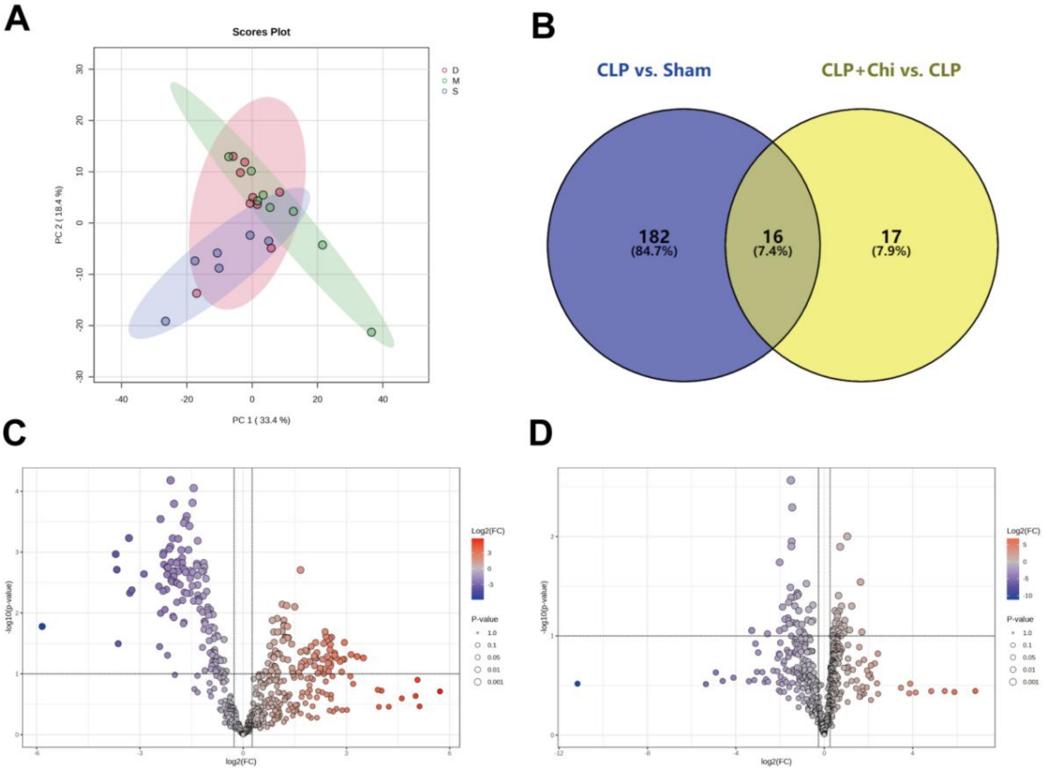

Figure S2: PCA, Venn, and volcano plots analysis in lipidomics. (A) PCA score plots and of rat

lung tissue lipidomics analysis between groups. (B) Venn diagram showing the differential lipid metabolites between the CLP vs. Sham and CLP+Chi 10 vs. CLP groups. (C, D) Volcano plots showing the differences between CLP vs. Sham and CLP+Chi 10 vs. CLP in LC-MS analysis with both positive and negative ion detection modes. Wherein, “S” represents the sham surgery group; “M” represents the CLP group; “D” represents the CLP+Chi 10 group.

Supplemental Figure S3

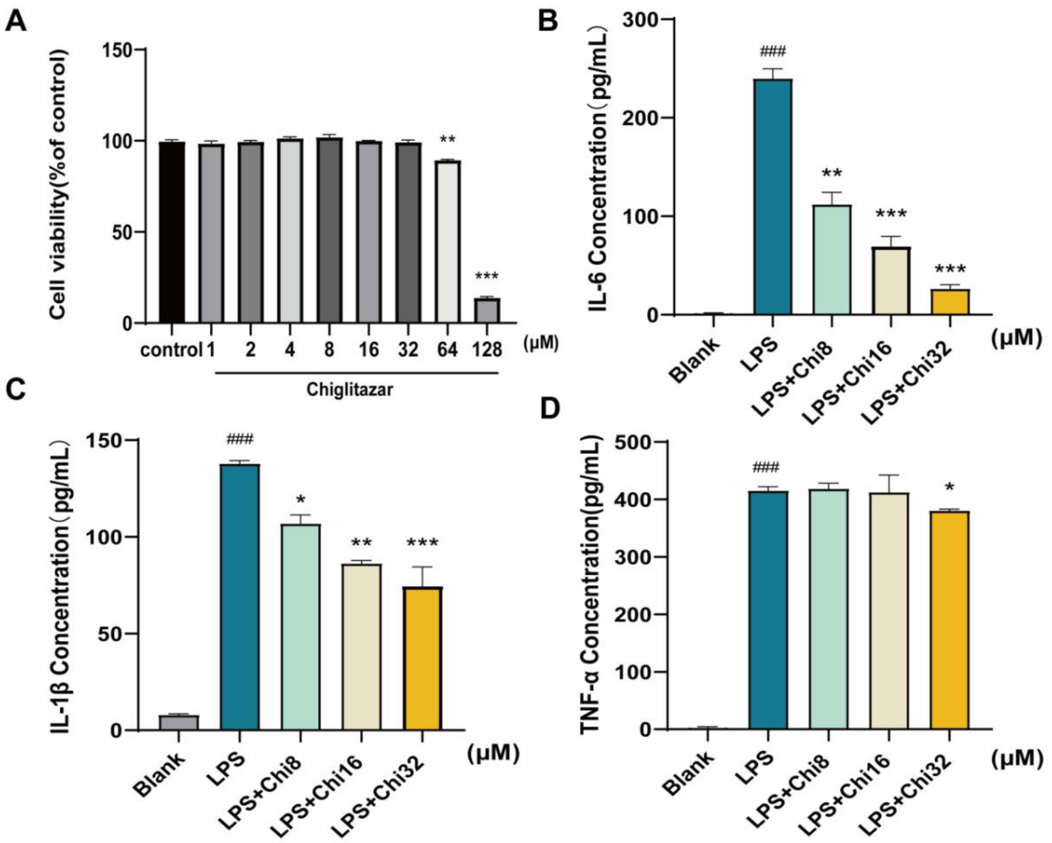

Figure S3: Cell viability and inflammatory factor levels of BEAS-2B cells incubated with different concentrations of chiglitazar. (A) Cell viability of BEAS-2B cells incubated with various concentrations of chiglitazar (1, 2, 4, 8, 16, 32, 64, and 128 μM) for 24 hours, $n = 3$. Levels of IL-6 (B), IL-1β (C), and TNF-α (D) in the culture supernatants of BEAS-2B cells treated with different concentrations of chiglitazar (8, 16, and 32 μM), $n = 3$. **$p < 0.01$,***$p < 0.001$, ###$p < 0.001$vs. Blank group; **$p < 0.05$,***$p < 0.001$vs. LPS group.

Supplemental Figure S4

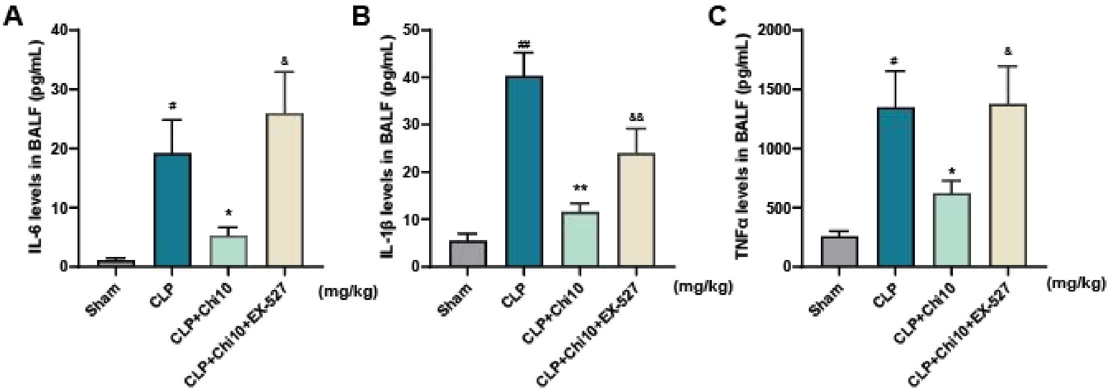

Figure S4: The effect of inhibitor EX-527 on the levels of inflammatory cytokines in BALF. Measures of IL-6 (A), IL-1β (B), and TNF-α (C) in the BALF of rats. $^{\#}p < 0.05$, $^{\#\#}p < 0.01$ vs. Sham group; $^{*}p < 0.05$, $^{**}p < 0.01$ vs. CLP group; $^{\&}p < 0.05$, $^{\&\&}p < 0.01$ vs. CLP+Chi10 group.

Table S1. Overview of experimental batches and group designs

Batch	Purpose	Groups	Sample Size (n)
1	Survival observation	Sham, CLP, CLP+Chi (2.5, 5, 10 mg/kg)	Sham: 6; CLP: 10; CLP+Chi: 10 per dose
2	Dose-dependent effects	Sham, CLP, CLP+Chi (2.5, 5, 10 mg/kg), CLP+Pio (10 mg/kg)	Sham: 6; CLP: 12; Others: 10
3	Multi-omics analysis	Sham, CLP, CLP+Chi (5, 10 mg/kg)	Sham: 6; CLP: 12; CLP+Chi: 10 per dose
4	SIRT1 inhibition on survival	Sham, CLP, CLP+Chi (10 mg/kg), CLP+Chi (10 mg/kg)+EX-527 (5 mg/kg)	Sham: 6; CLP: 10; Others: 10
5	EX-527 antagonism	Sham, CLP, CLP+Chi (10 mg/kg), CLP+Chi (10 mg/kg)+EX-527 (5 mg/kg)	Sham: 6; CLP: 12; Others: 10

Table S2. Differential lipid metabolites between CLP and Sham groups. Statistical significance was confirmed as $p < 0.05$ was considered as the threshold of significant difference. up/down: increase/decrease in the CLP group.

Metabolite	m/z	RT (min)	CLP vs.Sham	Mode
ChE(20:4)	690.6184	24.027	up	Positive
ChE(22:6)	714.6184	23.786	up	Positive
DG(16:0/16:1)	584.5249	18.15	down	Positive

DG(32:0)	570.5456	21.13	down	Positive
DG(32:1)	568.5299	20.383	down	Positive
LPC(17:0)	510.3554	5.074	up	Positive
LPC(18:1)	522.3554	4.699	up	Positive
PC(15:0/23:6)	806.5694	14.761	up	Positive
PC(32:0)	734.5694	16.495	up	Positive
PC(34:1)	760.5851	16.735	up	Positive
PC(36:1)	788.6164	18.394	up	Positive
PC(36:2)	786.6007	17.303	up	Positive
PC(38:3)	812.6164	17.811	up	Positive
PC(38:4)	810.6007	16.594	up	Positive
PC(38:5)	808.5851	15.397	up	Positive
PC(38:6)	806.5694	13.513	up	Positive
PC(38:7)	804.5538	15.651	up	Positive
PC(39:4)	824.6164	17.68	up	Positive
PC(40:4)	838.6320	18.251	up	Positive
PC(40:5)	836.6164	17.355	up	Positive
PC(40:6)	834.6007	15.724	up	Positive
SM(d34:1)	703.5749	13.777	up	Positive
SM(d42:2)	813.6844	19.318	up	Positive
SM(d42:3)	811.6688	18.356	up	Positive
TG(10:0/18:2/18:2)	788.6763	21.646	down	Positive
TG(12:0/12:0/18:3)	717.6028	21.749	down	Positive
TG(12:0/14:0/14:0)	712.6450	21.265	down	Positive
TG(12:0/14:0/17:3)	731.6184	21.875	down	Positive
TG(12:0/14:0/18:3)	745.6341	22.191	down	Positive
TG(14:0/13:0/14:1)	724.6450	21.591	down	Positive
TG(14:0/14:0/17:3)	759.6497	22.414	down	Positive
TG(14:0/14:0/18:3)	773.6654	22.638	down	Positive
TG(14:0/18:2/18:2)	844.7389	22.552	down	Positive
TG(14:1/14:1/14:1)	734.6293	21.158	down	Positive
TG(15:0/10:0/10:0)	642.5667	20.062	down	Positive
TG(15:0/12:0/14:0)	726.6606	21.975	down	Positive
TG(15:0/14:0/14:0)	754.6919	22.409	down	Positive
TG(15:0/14:0/15:0)	768.7076	22.222	down	Positive
TG(15:0/14:0/16:0)	782.7232	22.85	down	Positive
TG(15:0/14:0/16:1)	780.7076	22.044	down	Positive
TG(15:0/14:0/18:3)	787.6810	22.859	down	Positive
TG(15:0/15:0/16:0)	796.7389	22.661	down	Positive
TG(15:0/16:0/16:0)	810.7545	23.339	down	Positive
TG(15:0/16:0/16:1)	808.7389	22.481	down	Positive
TG(15:0/16:0/17:1)	822.7545	22.707	down	Positive
TG(15:0/16:0/18:1)	836.7702	22.919	down	Positive
TG(15:0/16:0/20:4)	841.7280	23.358	down	Positive

TG(15:0/16:1/16:1)	806.7232	22.124	down	Positive
TG(15:0/16:1/24:0)	920.8641	25.195	down	Positive
TG(15:0/18:1/22:6)	908.7702	22.63	up	Positive
TG(15:0/18:2/18:2)	858.7545	22.635	down	Positive
TG(15:0/18:2/20:4)	865.7280	22.988	down	Positive
TG(15:0/18:2/20:5)	863.7123	22.644	down	Positive
TG(15:0/18:2/22:6)	906.7545	22.404	up	Positive
TG(15:0/8:0/16:1)	696.6137	21.155	down	Positive
TG(15:1/12:0/14:1)	722.6293	21.155	down	Positive
TG(15:1/14:0/14:1)	750.6606	21.676	down	Positive
TG(15:1/14:0/16:1)	761.6654	22.323	down	Positive
TG(15:1/14:1/14:1)	748.6450	21.227	down	Positive
TG(15:1/14:1/16:1)	776.6763	21.756	down	Positive
TG(15:1/16:1/16:1)	804.7076	22.178	down	Positive
TG(15:1/16:1/18:2)	830.7232	22.331	down	Positive
TG(16:0/10:0/10:0)	656.5824	20.572	down	Positive
TG(16:0/12:0/14:0)	740.6763	21.839	down	Positive
TG(16:0/14:0/14:0)	768.7076	22.636	down	Positive
TG(16:0/14:0/16:1)	794.7232	22.281	down	Positive
TG(16:0/16:0/16:0)	824.7702	23.105	down	Positive
TG(16:0/16:0/16:1)	822.7545	23.147	down	Positive
TG(16:0/16:0/17:0)	838.7858	23.339	down	Positive
TG(16:0/16:0/18:1)	850.7858	23.167	down	Positive
TG(16:0/16:0/20:4)	855.7436	23.605	down	Positive
TG(16:0/16:0/20:5)	853.7280	23.194	down	Positive
TG(16:0/16:0/24:0)	936.8954	25.983	down	Positive
TG(16:0/16:1/16:1)	820.7389	22.337	down	Positive
TG(16:0/16:1/17:0)	836.7702	23.354	down	Positive
TG(16:0/16:1/17:1)	834.7545	22.955	down	Positive
TG(16:0/16:1/18:1)	848.7702	22.758	down	Positive
TG(16:0/16:1/19:4)	839.7123	22.956	down	Positive
TG(16:0/16:1/21:0)	892.8328	24.464	down	Positive
TG(16:0/17:0/18:1)	864.8015	23.851	down	Positive
TG(16:0/17:0/18:3)	843.7436	23.812	down	Positive
TG(16:0/17:0/20:4)	869.7593	23.872	down	Positive
TG(16:0/17:1/18:1)	862.7858	23.382	down	Positive
TG(16:0/18:1/20:5)	896.7702	22.962	up	Positive
TG(16:0/18:2/21:0)	918.8484	22.955	down	Positive
TG(16:0/18:2/22:4)	924.8015	23.064	up	Positive
TG(16:0/18:3/18:3)	851.7123	22.784	down	Positive
TG(16:0/20:4/20:5)	901.7280	22.732	up	Positive
TG(16:0/20:4/22:5)	929.7593	23.215	up	Positive
TG(16:0/20:4/22:6)	927.7436	22.947	up	Positive
TG(16:0/20:5/20:5)	899.7123	22.462	up	Positive

TG(16:0/20:5/22:6)	925.7280	22.615	up	Positive
TG(16:0/8:0/10:0)	628.5511	15.576	down	Positive
TG(16:0/8:0/8:0)	600.5198	18.463	down	Positive
TG(16:1/12:0/12:0)	710.6293	21.346	down	Positive
TG(16:1/12:0/14:0)	738.6606	21.832	down	Positive
TG(16:1/12:0/14:1)	736.6450	21.434	down	Positive
TG(16:1/13:0/14:0)	752.6763	22.045	down	Positive
TG(16:1/14:0/14:0)	766.6919	21.836	down	Positive
TG(16:1/14:0/14:1)	764.6763	21.908	down	Positive
TG(16:1/14:0/16:1)	792.7076	22.332	down	Positive
TG(16:1/14:0/17:3)	785.6654	22.479	down	Positive
TG(16:1/14:0/18:2)	818.7232	22.387	down	Positive
TG(16:1/14:1/14:1)	762.6606	21.517	down	Positive
TG(16:1/14:1/16:1)	790.6919	21.975	down	Positive
TG(16:1/16:1/16:2)	816.7076	22.136	down	Positive
TG(16:1/16:1/17:0)	834.7545	22.541	down	Positive
TG(16:1/16:1/17:1)	832.7389	22.576	down	Positive
TG(16:1/16:1/17:3)	811.6810	22.54	down	Positive
TG(16:1/16:1/18:1)	846.7545	22.788	down	Positive
TG(16:1/16:1/18:3)	825.6967	22.766	down	Positive
TG(16:1/16:1/20:5)	849.6967	22.533	down	Positive
TG(16:1/16:1/23:0)	918.8484	24.568	down	Positive
TG(16:1/16:1/24:0)	932.8641	24.925	down	Positive
TG(16:1/17:0/18:1)	862.7858	22.956	down	Positive
TG(16:1/17:1/18:1)	860.7702	22.996	down	Positive
TG(16:1/17:1/18:2)	858.7545	22.69	down	Positive
TG(16:1/8:0/10:0)	626.5354	18.547	down	Positive
TG(16:1/8:0/14:0)	682.5980	20.737	down	Positive
TG(16:1/8:0/18:3)	715.5871	21.331	down	Positive
TG(17:0/18:1/18:1)	890.8171	23.905	down	Positive
TG(17:0/18:1/22:5)	938.8171	23.303	up	Positive
TG(17:4/14:0/16:1)	783.6497	22.115	down	Positive
TG(18:0/16:0/16:0)	852.8015	23.587	down	Positive
TG(18:0/17:0/20:4)	914.8171	23.558	down	Positive
TG(18:0/18:0/22:6)	952.8328	23.988	up	Positive
TG(18:0/20:3/20:4)	933.7906	24.161	up	Positive
TG(18:0/20:4/22:6)	972.8015	22.962	up	Positive
TG(18:1/17:1/18:1)	888.8015	23.433	down	Positive
TG(18:1/18:1/19:3)	895.7749	23.888	down	Positive
TG(18:1/18:2/20:4)	922.7858	22.747	up	Positive
TG(18:1/18:2/20:5)	920.7702	22.616	up	Positive
TG(18:1/18:2/22:5)	948.8015	23.017	up	Positive
TG(18:1/20:4/22:4)	974.8171	22.955	up	Positive
TG(18:1/20:4/22:5)	972.8015	22.614	up	Positive

TG(18:1/20:4/22:6)	953.7593	23.083	up	Positive
TG(18:1/22:4/22:6)	998.8171	22.805	up	Positive
TG(18:1/22:5/22:6)	996.8015	22.506	up	Positive
TG(18:2/17:1/20:4)	908.7702	22.625	up	Positive
TG(18:2/18:2/18:2)	896.7702	22.509	up	Positive
TG(18:2/18:2/20:4)	920.7702	22.403	up	Positive
TG(18:2/18:2/21:3)	936.8015	22.853	up	Positive
TG(18:2/18:2/22:6)	944.7702	22.293	up	Positive
TG(18:2/18:2/23:0)	970.8797	24.739	down	Positive
TG(18:2/20:4/22:6)	951.7436	22.622	up	Positive
TG(18:2/22:6/22:6)	992.7702	22.085	up	Positive
TG(18:3/18:2/18:2)	877.7280	22.838	up	Positive
TG(18:4/12:0/13:0)	729.6028	21.539	down	Positive
TG(18:4/12:0/14:0)	743.6184	21.828	down	Positive
TG(18:4/12:0/15:0)	757.6341	22.042	down	Positive
TG(18:4/12:0/15:1)	755.6184	21.68	down	Positive
TG(18:4/13:1/16:1)	781.6341	21.755	down	Positive
TG(18:4/14:0/14:0)	771.6497	22.262	down	Positive
TG(18:4/14:0/14:1)	769.6341	21.902	down	Positive
TG(18:4/14:0/16:0)	799.6810	22.691	down	Positive
TG(18:4/14:0/16:1)	797.6654	22.336	down	Positive
TG(18:4/14:1/16:1)	795.6497	21.977	down	Positive
TG(18:4/15:0/16:0)	813.6967	22.93	down	Positive
TG(18:4/15:1/16:1)	809.6654	22.191	down	Positive
TG(18:4/16:0/16:0)	827.7123	23.149	down	Positive
TG(18:4/16:1/16:1)	823.6810	22.389	down	Positive
TG(18:4/16:1/17:1)	837.6967	22.583	down	Positive
TG(18:4/17:0/18:1)	867.7436	23.424	down	Positive
TG(18:4/8:0/10:0)	631.4932	18.546	down	Positive
TG(18:4/8:0/8:0)	603.4619	17.103	down	Positive
TG(19:1/16:0/18:2)	871.7749	24.414	down	Positive
TG(19:1/18:1/18:1)	916.8328	23.971	down	Positive
TG(20:5/18:2/20:4)	925.7280	22.403	up	Positive
TG(20:5/18:2/20:5)	923.7123	22.358	up	Positive
TG(20:5/18:2/22:6)	949.7280	22.295	up	Positive
TG(20:5/20:5/22:6)	971.7123	21.93	up	Positive
TG(22:5/18:2/18:2)	946.7858	22.305	up	Positive
TG(22:5/18:2/20:4)	970.7858	22.505	up	Positive
TG(22:5/18:2/22:6)	994.7858	22.229	up	Positive
TG(24:1/18:2/20:4)	1006.8797	24.195	up	Positive
TG(24:3/16:0/18:2)	937.8219	24.873	down	Positive
TG(25:0/16:1/16:1)	946.8797	25.317	down	Positive
TG(26:1/18:2/20:4)	1034.9110	25.011	up	Positive
TG(4:0/10:0/18:3)	577.4463	16.743	down	Positive

TG(8:0/10:0/10:0)	544.4572	14.281	down	Positive
TG(8:0/10:0/17:1)	640.5511	19.5	down	Positive
TG(8:0/10:0/18:1)	654.5667	19.714	down	Positive
TG(8:0/8:0/10:0)	516.4259	11.687	down	Positive
TG(8:0/8:0/18:3)	605.4776	18.467	down	Positive
TG(8:0/8:0/8:0)	488.3946	8.361	down	Positive
PE(34:0)	718.5392	9.34	up	Negative
FA(22:5)	329.2486	2.34	up	Negative
FA(22:4)	331.2643	2.31	up	Negative
FA(22:6)	327.233	1.75	up	Negative
FA(20:4)	303.233	1.9	up	Negative
FA(20:2)	307.2643	2.47	down	Negative
FA(20:3)	305.2486	2.14	down	Negative
FA(16:2)	251.2017	1.64	down	Negative
FA(26:0)	395.3895	7.78	up	Negative
FA(24:0)	367.3582	6.51	up	Negative
FA(21:0)	325.3112	4.52	up	Negative
FA(23:0)	353.3425	5.88	up	Negative
FA(22:0)	339.3269	5.18	up	Negative

Table S3. Differential lipid metabolites between CLP+Chi 10 and CLP groups. Statistical significance was confirmed as $p < 0.05$ was considered as the threshold of significant difference. up/down: increase/decrease in the CLP+Chi 10 group.

Metabolite	m/z	RT (min)	CLP+Chi10 vs.CLP	Mode
DG(32:0)	570.5456	21.13	up	Positive
LPC(18:0)	524.3711	5.671	down	Positive
LPC(18:1)	522.3554	4.699	down	Positive
LPC(20:4)	544.3398	4.013	down	Positive
PC(36:4)	782.5694	13.577	down	Positive
PC(38:4)	810.6007	16.594	down	Positive
TG(10:0/10:0/10:0)	572.4885	16.451	up	Positive
TG(10:0/10:0/18:3)	661.5402	20.635	up	Positive
TG(15:0/10:0/10:0)	642.5667	20.062	up	Positive
TG(15:0/14:0/15:0)	768.7076	22.222	up	Positive
TG(15:0/8:0/14:0)	670.5980	21.282	up	Positive
TG(16:0/14:0/16:1)	794.7232	22.281	up	Positive
TG(16:0/14:1/20:4)	842.7232	22.199	down	Positive
TG(16:0/16:1/16:1)	820.7389	22.337	up	Positive
TG(16:0/18:1/24:7)	931.7749	23.434	down	Positive
TG(16:1/12:0/12:0)	710.6293	21.346	up	Positive

TG(16:1/12:0/14:0)	738.6606	21.832	up	Positive
TG(16:1/8:0/18:3)	715.5871	21.331	up	Positive
TG(8:0/10:0/10:0)	544.4572	14.283	up	Positive
TG(8:0/8:0/18:3)	605.4776	18.467	up	Positive
FA(27:3)	403.3582	6.69	down	Negative
FA(26:1)	393.3738	6.59	down	Negative
FA(15:1)	239.2017	1.72	up	Negative
FA(28:7)	409.3112	5.87	down	Negative
FA(20:1)	309.2799	3.03	down	Negative
FA(14:1)	225.186	1.57	up	Negative
FA(18:3)	277.2173	1.72	down	Negative
FA(18:1)	281.2486	2.36	down	Negative
FA(18:2)	279.233	1.98	down	Negative
FA(22:5)	329.2486	2.34	down	Negative
FA(22:4)	331.2643	2.31	down	Negative
FA(22:6)	327.233	1.75	down	Negative
FA(20:4)	303.233	1.9	down	Negative
